# Supplementary figures and images for: Human immune response to primary cryptosporidiosis parallels murine infection models
Source: Infect Immun. 2026 Feb 4;94(3):e00701-25. doi: 10.1128/iai.00701-25 (PMC12974145; doi:10.1128/iai.00701-25)

**A**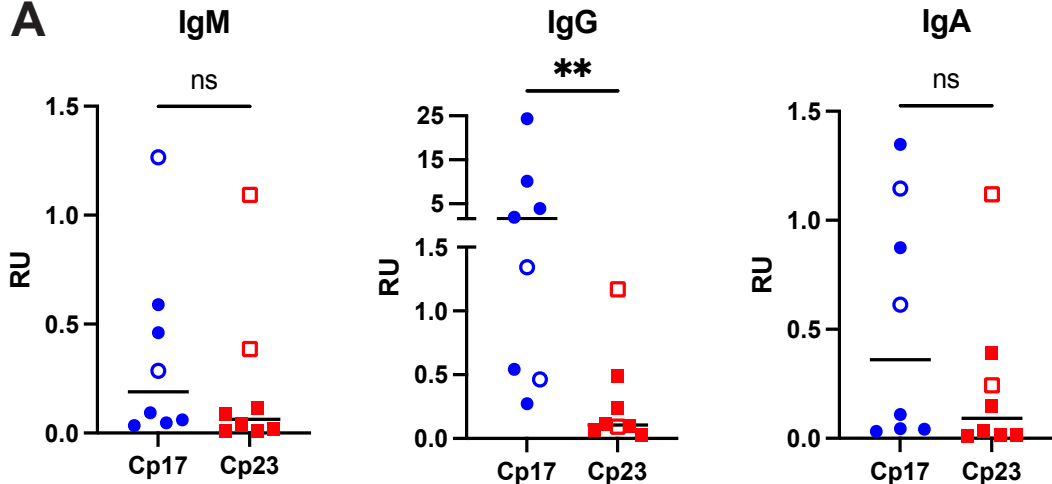**B**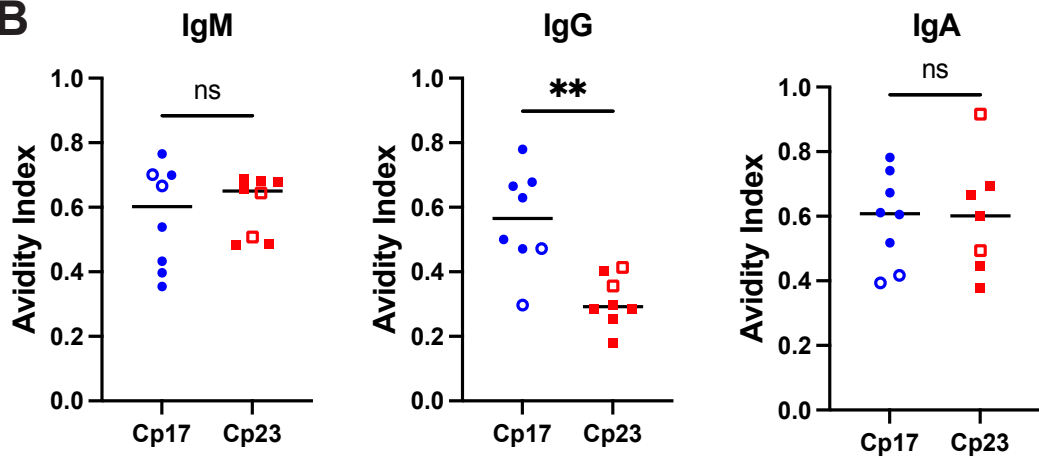

Supplement: Figure S1 — American adult compared to Bangladeshi child antibody avidity and quantity. [file iai.00701-25-s0001.pdf]

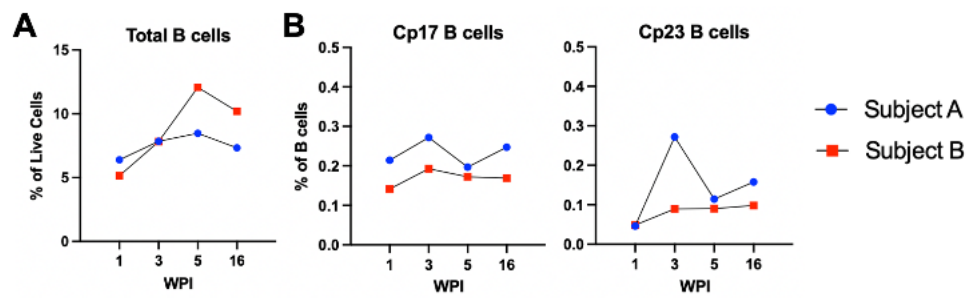

Supplement: Figure S2 — Total B cells. [file iai.00701-25-s0002.pdf]

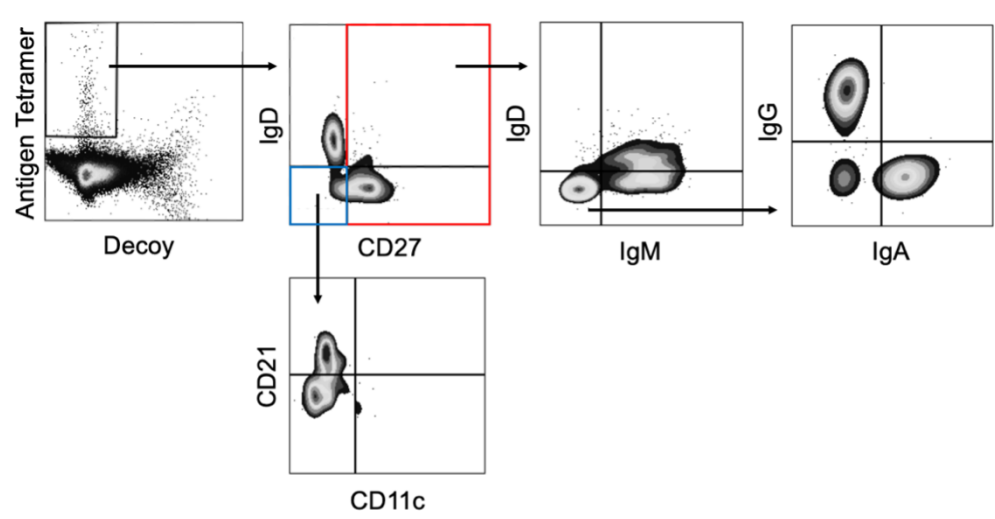

Supplement: Figure S3 — Flow gating strategy. [file iai.00701-25-s0003.pdf]

**A****Luminex PCA**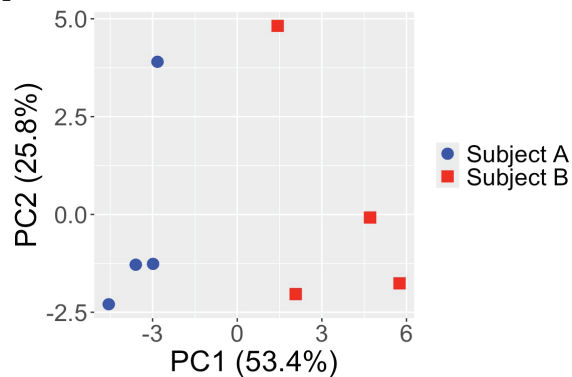**Luminex PCA**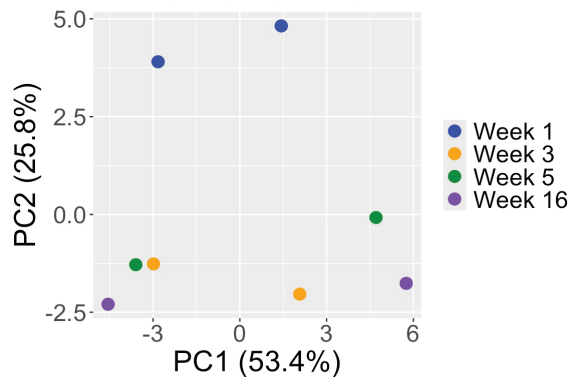**B****TGF $\alpha$** 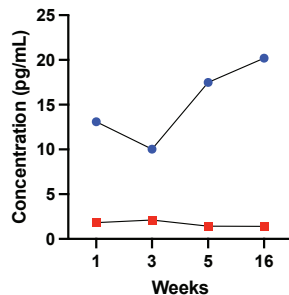**PDGF-AB/BB**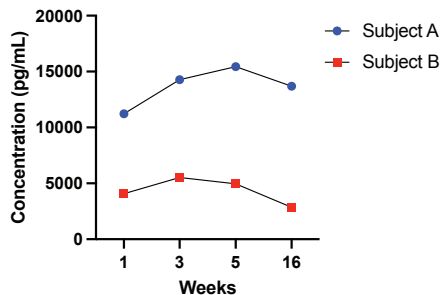**C****C-Reactive Protein**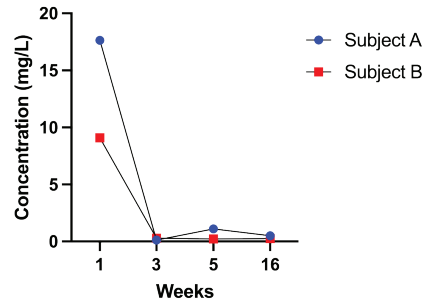

Supplement: Figure S4 — Cytokine PCA and PC1 cytokines. [file iai.00701-25-s0004.pdf]
