# Supplementary material for: Human immune response to primary cryptosporidiosis parallels murine infection models
Source: Infect Immun. 2026 Feb 4;94(3):e00701-25. doi: 10.1128/iai.00701-25 (PMC12974145; doi:10.1128/iai.00701-25)
Supplement: Supplemental material — Supplemental figure legends. [file iai.00701-25-s0005.docx]

**Supplementary Figure 1. American adults mount a similar antibody response to primary *Cryptosporidium* infection compared to Bangladeshi children. A)** Antibody quantity and **B)** antibody avidity to *Cryptosporidium spp*. proteins, Cp17 and Cp23, 3 weeks after an individual’s first *Cryptosporidium* infection. Closed symbols indicate Bangladeshi children sampled at one or two years of age. Open symbols indicate American adults. RU = relative units. Antibody quantity data were analyzed using the Mann-Whitney U test; antibody avidity data were analyzed using a Student’s T-Test. Significance codes: ns = P > 0.05, ** = P ≤ 0.01.

**Supplementary Figure 2. Total measure of B cells increased from start of infection while antigen-specific B cells peak at week 3. A-B)** Analysis of B cell populations in PBMCs acquired at weeks 1, 3, 5, and 16. **A)** Percentage of total B cell populations (CD3- CD14- CD16-, CD20+ and/or CD19+) out of all live cells. **B)** Percentage of total Cp17 B cells (left) and Cp23 B cells (right) (antigen tetramer+ decoy tetramer-) out of all B cells.

**Supplementary Figure 3. Example gating strategy for double negative (DN) B-cell subtypes and class-switched CD27+ B cells.** Double negative B cells (IgD- CD27-, **blue**) were further divided into subtypes based on CD21 and CD11c expression: CD21- CD11c- (DN3), CD21+ CD11c- (DN1), CD21- CD11c+ (DN2), and CD21+ CD11c+ (DN4). For isotype analysis, CD27+ B cells (**red**) were first gated by IgD and IgM expression. Cells negative for both IgD and IgM were subsequently classified by expression of IgG and IgA.

**Supplementary Figure 4. Subject-to-subject variation and time from infection shape circulating cytokine profiles during acute *Cryptosporidium* infection and convalescence. A)** Visualized principal component analysis (PCA) of plasma Luminex bead-based cytokine quantification color coded by subject (left) and timepoint (right). **B)** Quantification of cytokines by Luminex bead-based assay over time that contribute most to PC1. **C)** Quantification of C-reactive protein generated by enzyme-linked immunosorbent assay (ELISA).
